# Using novel micropore technology combined with artificial intelligence to differentiate *Staphylococcus aureus* and *Staphylococcus epidermidis*

**DOI:** 10.1038/s41598-024-55773-4

**Published:** 2024-03-24

**Authors:** Ayumi Morimura, Masateru Taniguchi, Hiroyasu Takei, Osamu Sakamoto, Norihiko Naono, Yukihiro Akeda, Daisuke Onozuka, Jumpei Yoshimura, Kazunori Tomono, Satoshi Kutsuna, Shigeto Hamaguchi

**Affiliations:** 1 Department of Infection Control and Prevention, Graduate School of Medicine, Osaka University, 2-2 Yamadaoka, Suita, Osaka 565-0871 Japan; 2 The Institute of Scientific and Industrial Research, Osaka University, 8-1 Mihogaoka, Ibaraki, Osaka 567-0047 Japan; 3 Aipore Inc., 26-1 Sakuraoka-cho, Shibuya-ku, Tokyo, 150-8512 Japan; 4 Department of Bacteriology I, National Institute of Infectious Diseases, 1-23-1 Toyama, Shinjuku-ku, Tokyo, 162-8640 Japan; 5 Department of Oral Microbe Control, Graduate School of Medicine, Osaka University, 2-2 Yamadaoka, Suita, Osaka 565-0871 Japan; 6 Department of Traumatology and Acute Critical Medicine, Graduate School of Medicine, Osaka University, 2-15 Yamadaoka, Suita, Osaka 565-0871 Japan; 7 Osaka Institute of Public Health, 1-3-3 Nakamichi, Higashinari-ku, Osaka, 537-0025 Japan; 8 Division of Infection Control and Prevention, Osaka University Hospital, 2-15 Yamadaoka, Suita, Osaka 565-0871 Japan; 9 Division of Fostering Required Medical Human Resources, Center for Infectious Disease Education and Research (CiDER), Osaka University, 2-2 Yamadaoka, Suita, Osaka 565-0871 Japan; 10 Department of Transformative Analysis for Human Specimen, Graduate School of Medicine, Osaka University, 2-2 Yamadaoka, Suita, Osaka 565-0871 Japan

**Keywords:** Microbiology techniques, Clinical microbiology, Infectious-disease diagnostics, Computational nanotechnology, Biomedical engineering, Machine learning

## Abstract

Methods for identifying bacterial pathogens are broadly categorised into conventional culture-based microbiology, nucleic acid-based tests, and mass spectrometry. The conventional method requires several days to isolate and identify bacteria. Nucleic acid-based tests and mass spectrometry are relatively rapid and reliable, but they require trained technicians. Moreover, mass spectrometry requires expensive equipment. The development of a novel, inexpensive, and simple technique for identifying bacterial pathogens is needed. Through combining micropore technology and assembly machine learning, we developed a novel classifier whose receiver operating characteristic (ROC) curve showed an area under the ROC curve of 0.94, which rapidly differentiated between *Staphylococcus aureus* and *Staphylococcus epidermidis* in this proof-of-concept study. Morphologically similar bacteria belonging to an identical genus can be distinguished using our method, which requires no specific training, and may facilitate the diagnosis and treatment of patients with bacterial infections in remote areas and in developing countries.

## Introduction

*Staphylococcus aureus* is one of the most important pathogens in humans, causing numerous infections ranging from furuncles to infective endocarditis^1,2^. *S. aureus* is a leading cause of nosocomial infections, such as catheter-related bloodstream infection, prosthetic joint infection, prosthetic valve endocarditis, and surgical site infection^1,2^. Another significant bacterium of the genus Staphylococcus, *Staphylococcus epidermidis*, is one of the most commonly found coagulase-negative staphylococci (CNS) and is predominant in human skin microbiota^3,4^. It causes various nosocomial opportunistic infections, particularly those associated with indwelling devices^5^, similar to *S. aureus* in the sense that both bacteria causes internal medical device infection^6,7^. Generally, *S. epidermidis* is less virulent than *S. aureus* but can be critical, particularly in causing bacteremia^8,9^. When a blood culture is reported to be positive for *Staphylococcus* species preliminarily in the clinical setting, clinicians must immediately respond to two questions, namely, whether the bacteria is *S. aureus* or CNS and whether it is a contaminant^10^. However, there are no rapid and reliable bedside assessment tools to distinguish *S. aureus* from CNS bacteria.

Today, the three general direct bacterial identification methods are as follows: conventional culture-based microbiology; nucleic acid-based tests, such as polymerase chain reaction (PCR) and 16S ribosomal RNA sequencing; and matrix-assisted laser desorption ionization-time-of-flight mass spectrometry (MALDI-TOF MS)^11,12^. Despite being well-established, the conventional method has disadvantages. First, not all bacteria can grow in the laboratory and, even if they are culturable, it is unclear to what extent culture growth is dependant on the environment (e.g., temperature, atmosphere, and medium)^13^. Several days to weeks are required to grow, isolate, and identify bacteria. Nucleic acid-based tests and MALDI-TOF MS are relatively rapid and reliable. However, PCR requires primers designed for each targeted bacterium, and 16S ribosomal RNA sequencing requires a sequencer and database reference, with both methods taking several hours to days to complete. MALDI-TOF MS cannot be directly used in clinical samples or to distinguish several pathogens (e. g. *Shigella* spp. vs *Escherichia coli*^14^, *Acinetobacter* non-*baumanii* species^15^). Additionally, it requires expensive equipment. Culture methods and nucleic-acid-based tests require trained technicians. Therefore, it is necessary to establish a novel, rapid, inexpensive, and user-friendly technique for the identification of bacteria.

Micropore devices can be used for optical cell detection and counting^16^. Furthermore, machine learning is useful for predicting antimicrobial resistance in bacteria identified using other methods^17^. In our study, we developed a disposable single-use micropore module for bacterial detection. The module has a pore of 3 μm diameter on a silicon film with two flow channels and electrodes. This module is easily handleable because the flow channels automatically absorb sample fluids by capillary action and needs no special techniques. When a single particle (e. g. a bacterium) move through the micropore, we can detect the change of ionic current. This change of ionic current varies depending on the particles. The combination of nanopore or micropore technology and machine learning has enabled the rapid detection and identification of microorganisms^18,19^. *Escherichia coli* (gram-negative) has been previously distinguished from *Bacillus subtilis* (gram-positive)^19^, which are microbiologically different.

Here, in this proof-of-concept study, using a micropore-based device assisted with artificial intelligence (AI), we as a first step aimed to develop a simple method to rapidly distinguish morphologically similar *S. aureus* from *S. epidermidis* that belong to an identical genus, to demonstrate the usefulness of AI-assisted diagnosis in clinical microbiology.

## Results

### A micropore device

**Figure 1:**
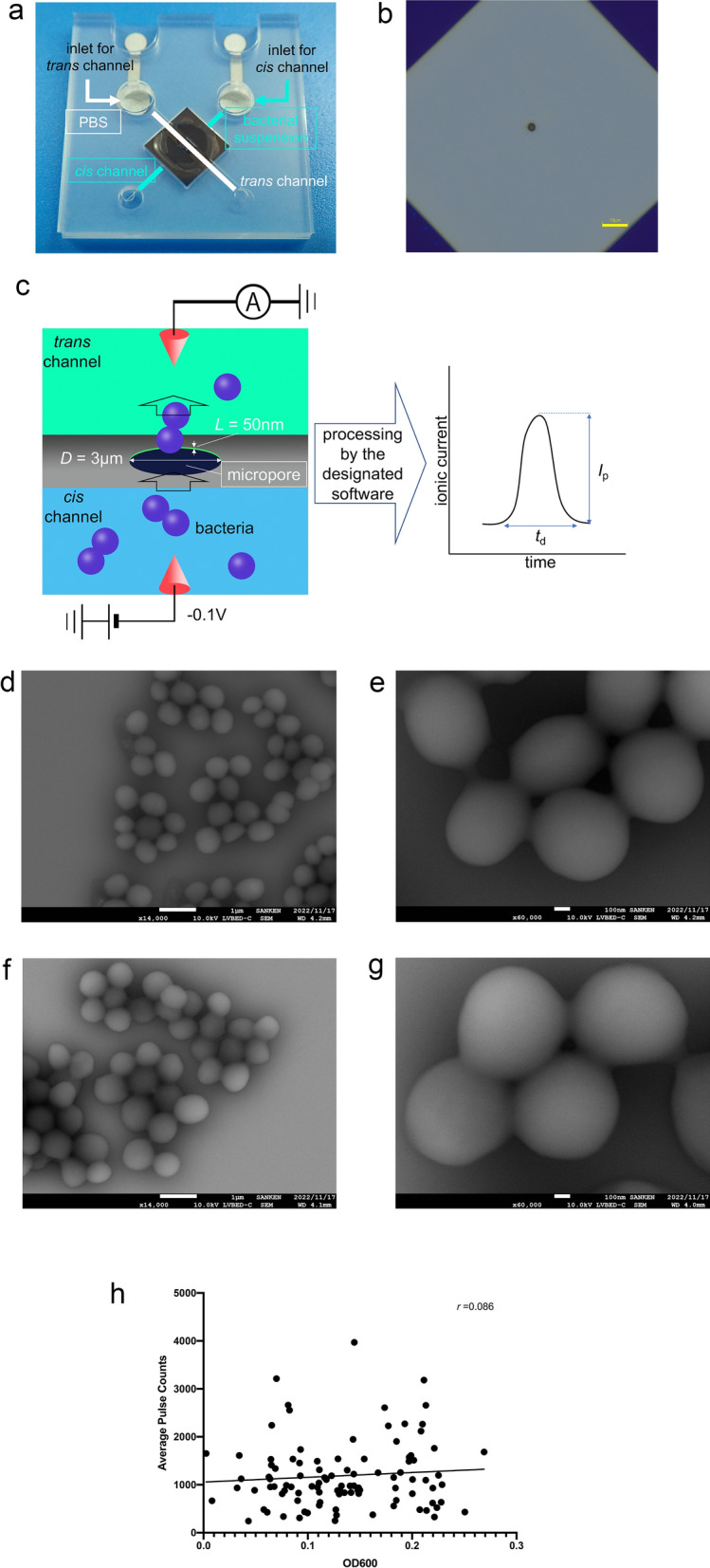
**(a)** The structure of the micropore module. The micropore module is 25 mm^2^ in size and 0.5 mm thick. The bacterial suspension is introduced from the *cis* channel, and PBS is introduced from the *trans* channel. (**b**) An optical image of the pore of the module. The optical microscopic examination of the micropore module suggests that the 3 μm diameter micropore is in the centre of the silicon substrate. (**c**) A schema of the micropore. When the bacteria pass through the micropore, the ionic current decreases because of obstruction in flowing ions. The processing software obtains the change in ionic current as a waveform. (**d**) Scanning electron microscope (SEM) observation of *S. epidermidis* ×14,000. (**e**) SEM observation of *S. epidermidis* ×60,000. **(f**) SEM observation of *S. aureus* ×14,000. (**g**) SEM observation of *S. aureus* ×60,000. **(h**) A scatter diagram of the OD600 of the bacterial suspensions and their average pulse counts. No correlations were observed between the OD600 and pulse counts of the bacterial suspensions.

Micropores (3 µm in diameter) were fabricated on a 50 nm-thick silicon nitride film cast on a silicon substrate. The silicon substrate was sandwiched between a 25 mm × 25 mm 0.5-mm-thick plastic channel. This structure is termed “a micropore module” or “a module” (Fig. 1a, b). Bacterial suspensions (18 µL) in 1× phosphate buffered saline (PBS) and PBS (15 µL) were introduced into the *cis* and *trans* chambers, respectively. We measured ionic current–time waveforms through applying a voltage of −0.1 V between the Ag/AgCl electrodes placed in the flow channel. When the bacteria passed through the micropores, the ionic current decreased owing to obstruction due to the flowing ions (Fig. 1c). *S. aureus* and *S. epidermidis* are spherical in shape with a diameter ranging from 0.6 to 1.0 µm under a low vacuum of 98 hPa. We observed no specific differences in the bacterial structures through scanning electron microscopy (SEM) under the culture conditions (Fig. 1d–g). The waveforms were collected in the data server and analysed, as described in the “Methods” section. The number of available waveforms for the bacterial suspensions was independent of their optical densities measured at a wavelength of 600 nm (OD600) (Fig. 1 h). Therefore, sufficient bacteria were present in the suspensions regardless of their OD600 values.

### Bacterial movement and ionic current

**Figure 2:**
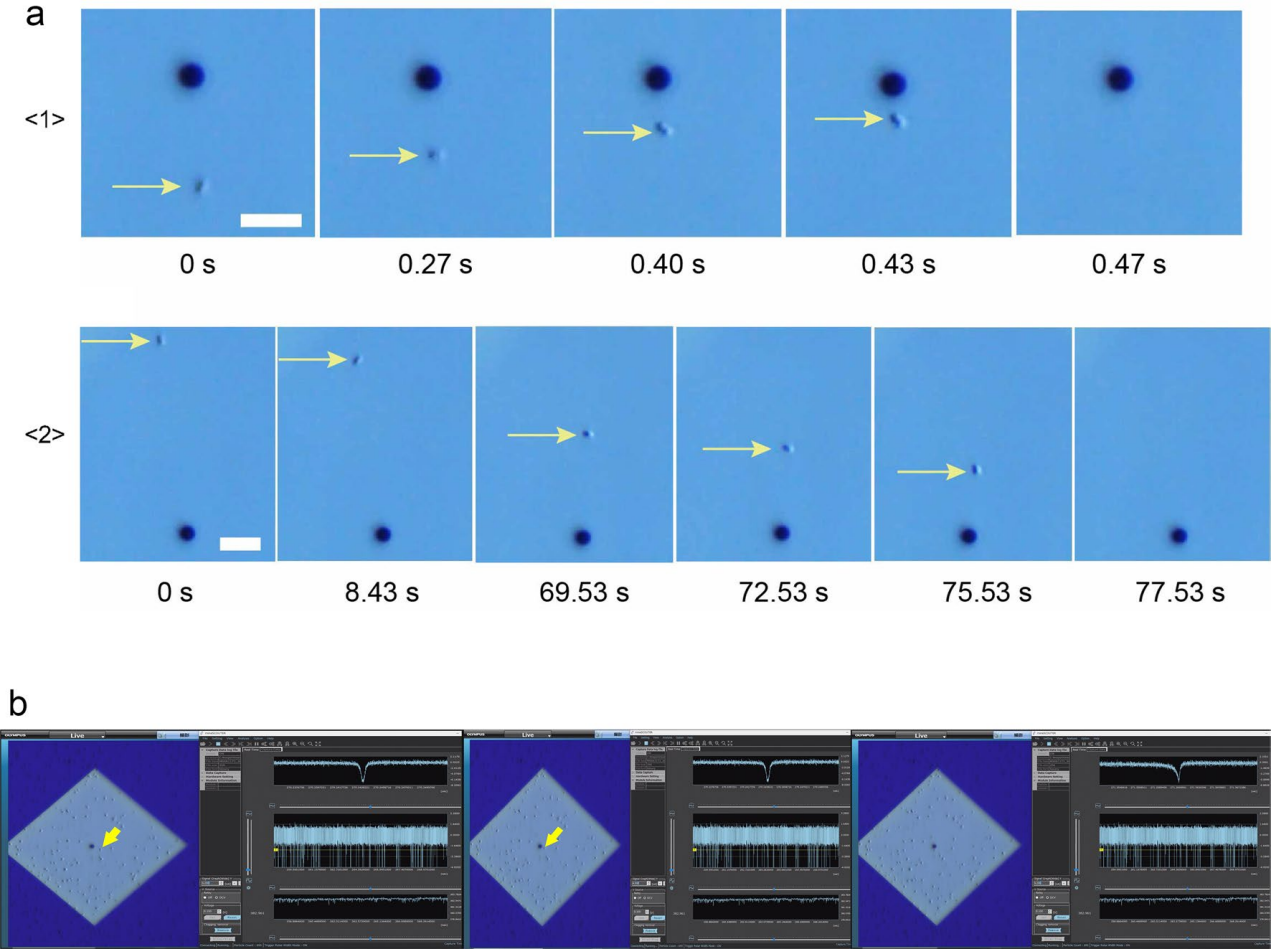
(**a**) Optical microscope images (scale bar = 10 µm) of *S. epidermidis* (small dot indicated by yellow arrow) being drawn towards and pulled into a micropore (3 µm black circles): <1**> **the bacterium near (within 15 µm radius) the micropore is pulled in at a fast rate; <2**> **the trajectory of a distant bacterium being drawn towards the micropore until it is pulled in. (**b**) Actual display screen during the measurement. The moment at which the bacteria (yellow arrow) is being sucked into the micropore. Its waveform is denoted on the right top column of the right-side window. The waveforms are indicated at three different time scales using the waveform viewer software.

Ionic current measurements were performed on *S. aureus* and *S. epidermidis* cultures using micropore devices placed under an optical microscope. The base current was approximately 0.4 µA. Using the equations for access resistance (1/*κD*) and micropore resistance (4*L*/π*κD*^2^), the base current was estimated to be 0.5 nA when the ionic conductivity (*κ*) of 1× PBS was 1.61 Sm^−1^, the average diameter of the micropores was *D* = 3 µm, and the thickness of the micropores was *L* = 50 nm. The ionic current obtained was consistent with the theoretically estimated ionic current. With application of a voltage of −0.1 V, a single bacterium (black spot indicated by yellow arrow, Fig. 2a) was pulled into the micropore. Bacteria within an approximate radius of 15 µm from the micropore were pulled in at an accelerated rate as they approached the micropore (Fig. 2a $\langle 1\rangle$). When one bacterium passed through the micropore, we observed a single ionic current–time waveform, and the ionic current did not change until it entered the micropore. Bacteria within an approximate radius of 40 µm from the micropore were drawn to the micropore by Brownian motion (Fig. 2a$\langle 2\rangle$). The duration of Brownian motion was > 1 min while approaching the micropores. When a bacterium reached an approximate 15 µm radius from the micropore threshold, it was rapidly pulled into the micropore, which suggested it was being pulled under the influence of electric forces.

Negatively charged bacteria were subjected to diffusion and electric forces between the electrodes. The radius (*r*) from the centre of the micropore at which the bacteria were trapped by the electric field is denoted by the equation:

$$r = d^{{2}} \mu \Delta V/{8}hD$$

where *d*, *µ*, and *ΔV* are the diameter of the micropore, the mobility of the bacteria, and applied voltage, respectively, and *h* and *D* are the thickness of the micropores and diffusion constant of the bacteria, respectively. The bacterial mobility and diffusion constants were approximately − 10^−8^ mV^−1^ s^−1^ and 10^−9^ m^2^ s^−1^, respectively. The experimentally observed *r* = 15 µm was relatively close to the theoretically predicted *r* = 22.5 µm. When the bacteria did not pass through the micropores, the ionic current remained constant at the base current, which was correlated with a non-event (Supplementary Material: the captured video of the actual screen during the measurement of the ionic currents of the bacteria. The optical microscopic image is shown on the left side, and the waveforms of ionic currents are shown on the right side). When a bacterium passed through the micropore, we obtained a spike-shaped ionic current–time waveform corresponding to one bacterium (Fig. 2b). The combination of optical microscopy and ionic current measurements demonstrated a correlation between the movement of a single bacterium and the ionic current–time waveform.

### Creating the classifier

**Figure 3:**
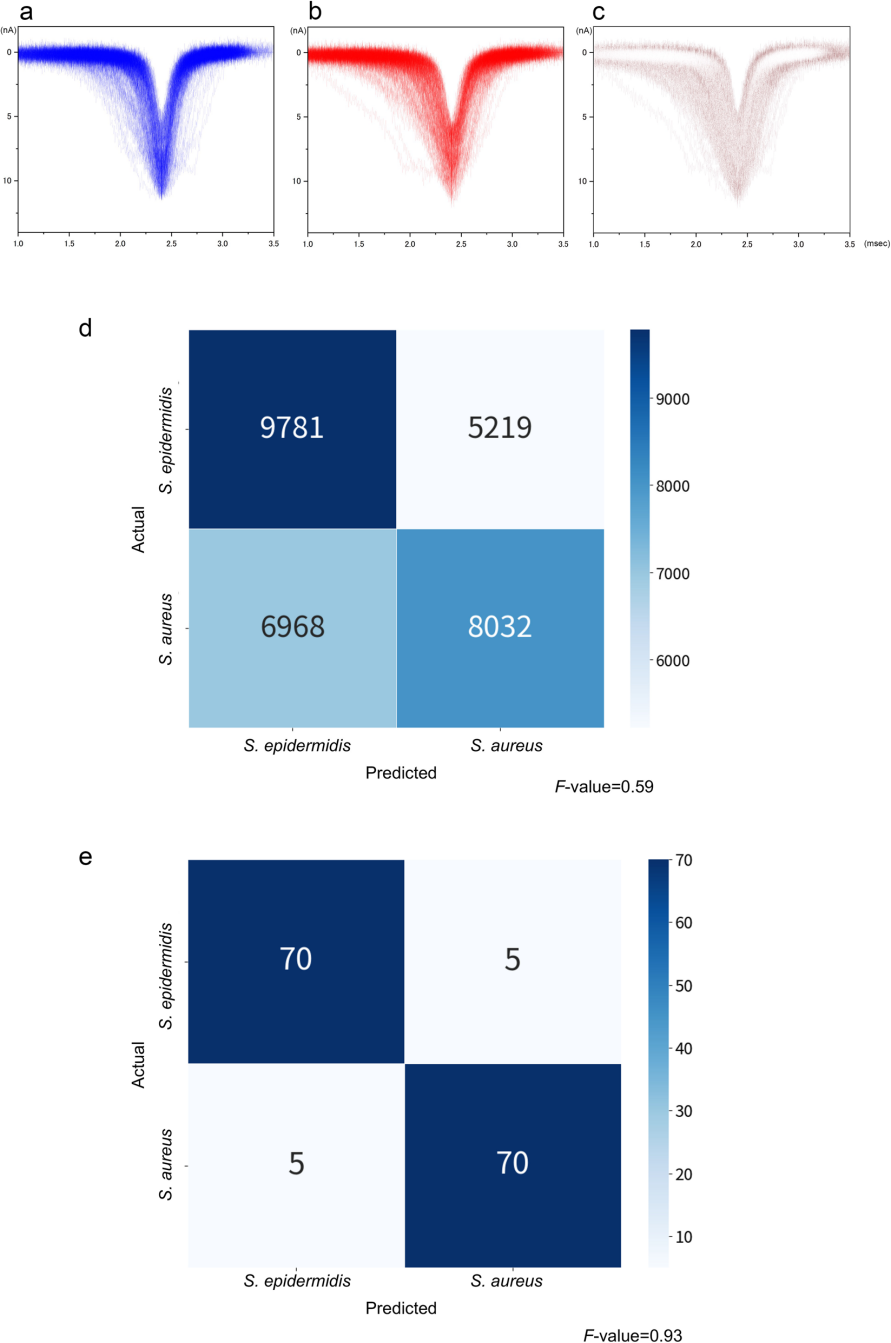
(**a**) Histograms of *I*_*p*_ and *t*_*d*_ of *S. epidermidis.* (**b**) Histograms of *I*_*p*_ and *t*_*d*_ of *S. aureus.* (**c**) Differences in the absolute value of the histograms of *I*_*p*_ and *t*_*d*_ between *S. epidermidis* and *S. aureus.* (**d**) Confusion matrix of all isolates. (**e**) Confusion matrix of the assembly machine learning results.

Spike-shaped waveforms are characterised by a maximum current value (*I*_p_) and current duration (*t*_d_) (Fig. 1c). The histograms of *I*_p_ and *t*_d_ nearly overlapped completely (Fig. 3a, b). Similar *I*_p_ levels reflected a small difference in the size between the two bacterial species. It was difficult to distinguish between them using these histograms. However, we observed differences in the shapes of the waveforms between the bacteria, suggesting distinguishable features (Fig. 3c). We used machine learning for identifying the waveform features such that the waveforms obtained could be used to identify the two species.

Fifty isolates of *S. aureus* and *S. epidermidis* were used for ionic current measurements. Each bacterial isolate was measured in triplicate using a micropore device, and each measurement lasted for 3 min. The 15,000 waveforms obtained from the measurements were provided as inputs for the machine-learning training set. The accuracy of differentiating between the two species in a single waveform was *F*-value = 0.59, which exceeded the *F*-value = 0.5 for random discrimination (Fig. 3d). In this single-waveform learning, it is determined which species each single waveform belongs to. The *F*-value is denoted by the harmonic mean of the sensitivity and precision, 2/(1/sensitivity + 1/precision), as follows:

$$Sensitivity= \frac{True \, Positive}{True \, Positive+False \, Negative}$$

$$Precision=\frac{True \, Positive}{True \, Positive+False \, Positive}$$

$$F-measure=\frac{2\times Sensitivity \, \times \, Precision}{Sensitivity \, + \, Precision}$$

For *S. epidermidis*, the confusion matrix yielded a sensitivity and precision of 9781/(9781 + 5219) = 0.65 and 9781/(9781 + 6968) = 0.58, respectively. This resulted in an *F*-value of 0.62. Similarly, the sensitivity and precision for *S. aureus* were 0.61 and 0.54, respectively, yielding an *F*-value of 0.57. The overall adopted *F*-value was the average of the *F*-values obtained for the two species. We determined the species-level accuracy of the bacterial identification on an isolate-by-isolate basis using machine learning. Of the 50 bacterial isolates, 25 were selected randomly as the training set for each species, respectively. We determined the training set yielding the highest *F*-value. Using the selected training set, we performed assembly learning to develop a classifier to determine whether a bacterial isolate belonged to *S. aureus* or *S. epidermidis*. In this assembly learning, single waveforms of one isolate/strain are treated as an aggregated data. Therefore, this assembly learning is an isolate/strain-focused learning. In contrast to the single-waveform learning, it is determined which species the aggregated data of each isolate/strain belongs to. In addition, the assembly machine learning uses the entire distribution of waveforms in each species, in addition to independent parameters (*I*_p_, *t*_d_, current vector, and time vector). The *F*-value was 0.93 (Fig. 3e).

### Distinguishing bacterial pathogens

**Figure 4:**
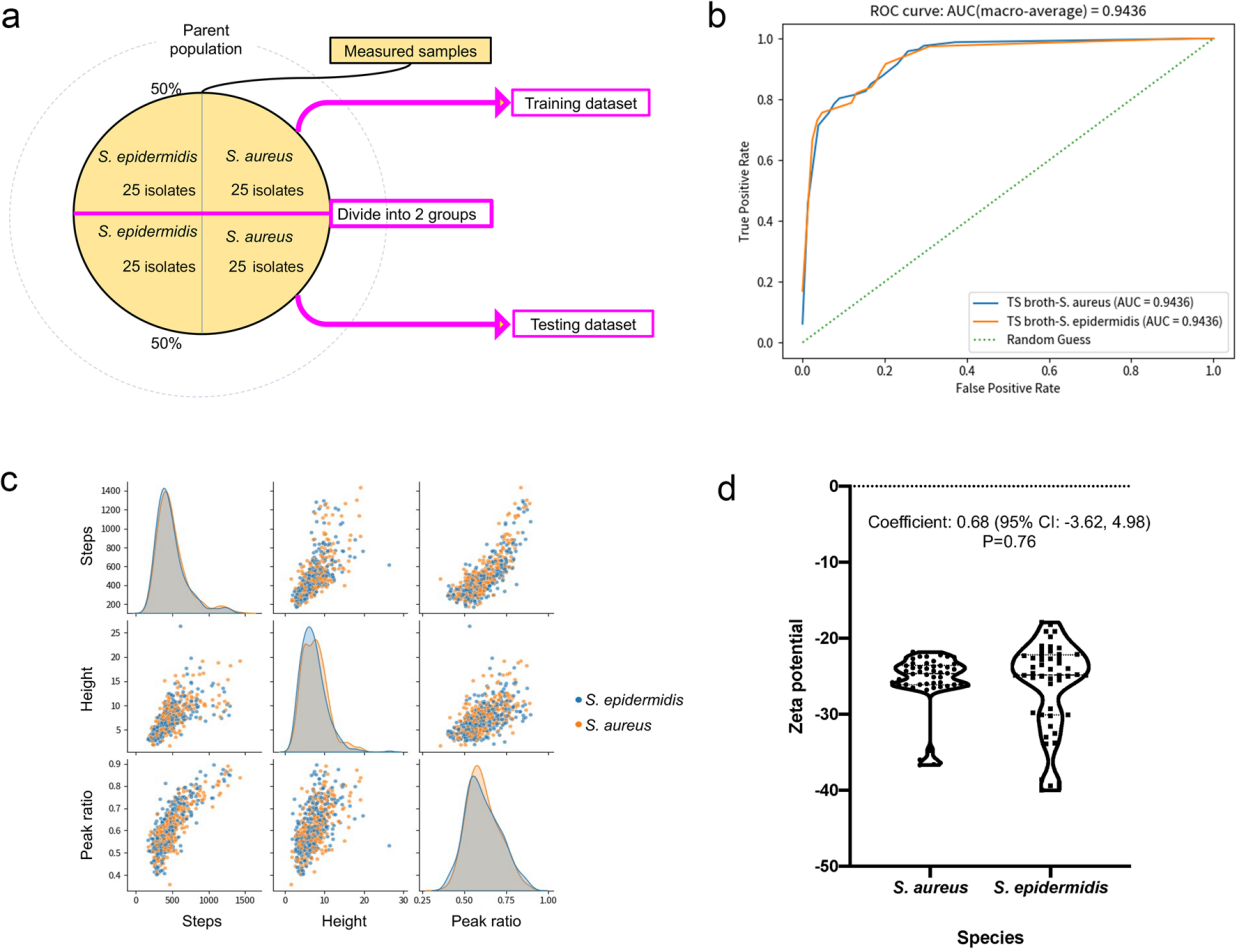
**(a)** Hold-out method. We employed a hold-out method for machine learning that splits data into the following two groups: a training dataset and a testing dataset. (**b**) Receiver operating characteristic curve of the classifier. (**c**) Characteristic distribution of the waveform. The pulse data are acquired 250,000 times per min. ‘Steps’ denote the number of data points acquired, ‘Height’ denotes the current value of the pulse, and ‘Peak ratio’ denotes the location of the peak of the pulse, when the left edge of the pulse is 0 and the right edge of the pulse is 1. (**d**) Zeta potential distribution of the bacteria. The dashed line denotes the median, and the dotted line denotes the quartiles. No statistically significant difference was noted between the two species.

The classifiers created during the machine learning process were used to distinguish the remaining 25 bacterial isolates (Fig. 4a) and the additional ATCC standard strains. Each isolate was assessed by the classifier for three measurements, and two or three correct responses from the three trials were regarded as the final correct answers for the isolate or strain.

The area under the receiver operating characteristic curve (AUROC) was 0.94 (> 0.9), demonstrating that the trained classifier could distinguish *S. aureus* from *S. epidermidis* at a high accuracy (Fig. 4b)^20^. The sensitivity and specificity for *S. aureus* detection were 96.4% and 80.8%, respectively, with an accuracy of 88.9% (Table 1). The positive agreement was 84.4%, and the negative agreement was 95.5% (Table 1).

**Table 1:** Final analysis of the isolates and strains using the classifier.

	*S. aureus* testing dataset isolates + ATCC 6538 + LAC (USA 300) + MW2 (n = 28)	*S. epidermidis* testing dataset isolates + ATCC 12228 (n = 26)
Determined to be *S. aureus* (n = 32)	27	5
Determined to be *S. epidermidis *(n = 22)	1	21

Accuracy, 88.9%; sensitivity for *S. aureus,* 96.4%; specificity *for S. aureus,* 80.8%; positive agreement, 84.4%; negative agreement, 95.5%.

Ionic current–time waveforms consist of information in relation to the size, shape, and surface charges of bacteria passing through the micropores. Micropore measurements demonstrated few statistically significant differences in the size and shape of *S. aureus* and *S. epidermidis* (Fig. 4c).

The Zeta potential affects the ionic current of the particles measured using the micropore device, and indicates the electrical charge of the surface layer of the particles^21^. Zeta potentials of the *S. aureus* and *S. epidermidis* isolates were measured using a Zetasizer™ (Malvern Instruments, Worcestershire, UK)^22^. The surface charges of the two species indicated that they were negatively charged, with Zeta potentials > −20 mV. While the Zeta potential range of *S. epidermidis* was greater than that of *S. aureus* and the distribution pattern was not completely the same, there were no significant difference between the Zeta potentials of *S. aureus* and S. *epidermidis* (Fig. 4d). Machine learning used surface charge differences between the two bacteria to distinguish between the species in reference to the entire distribution pattern of the features, of which difference is too subtle to statistically detect. The ion current–time waveform provides information on the volume, structure, and surface charge of bacteria passing through micropores. The machine learning, which inputs the shape of the ion current–time waveform as a feature, is considered to distinguish differences in surface charge.

## Discussion

The development of this bacterial identification technology, which combines micropore devices and machine learning, is a significant breakthrough because it is independent of biochemistry, molecular biology, and mass spectrometry. Bacteriologically distinct species have been previously distinguished using this method^19^. Herein, bacteria, namely *S. aureus* and *S. epidermidis*, belonging to an identical genus, could be distinguished using a combination of improved micropore devices and the assembly machine learning that used the entire set of features in each dataset, in addition to the classical parameters. This technique has three major benefits, as follows: rapidness, high accuracy, and low cost. First, the classifier required only a few seconds to identify the bacteria. Second, the AUROC was 0.94, the sensitivity for distinguishing *S. aureus* from *S. epidermidis* was 96.4%, the positive agreement was 84.4%, and the negative agreements was 95.5%, which highlights the usefulness of applying this technology to clinical medicine. From the viewpoint of antimicrobial stewardship, the rapid identification of infecting bacteria facilitates immediate administration of appropriate narrow spectrum antibiotics for infected patients. This is likely to help overcome antimicrobial resistance, which is a considerable threat to public health worldwide^23^. Third, this is a low-cost technique with respect to device and training operators. After mass production of the modules and detectors, the total running cost was estimated to be a few dollars per examination. The application of fluid samples to the module is simple because the fluid automatically fills the flow channel by capillary action. Therefore, operators can use the device without requiring special training.

A limitation of this study was that relatively few isolates were used for the dataset. However, we used 50 clinical isolates of *S. aureus* and *S. epidermidis* with 175,878 waveforms to develop the classifier. Moreover, the testing dataset included 50 clinical isolates and ATCC standard strains. We intend to gather more isolates to increase accuracy. Additionally, an adequate number of bacteria is required to create high-performance classifiers. This requirement can be a barrier in the development of classifiers using clinical samples with low numbers of bacteria. Furthermore, it is difficult to determine which feature AI predominantly focusses on during decision making. The cell wall anchored proteins of *S. aureus* and *S. epidermidis* is reported to be different^24,25^, so the manipulation of cell wall anchored proteins can be the target of further investigation to clarify the key features. In this study, it is certain that the AI considers the entire dataset of features. The assembly machine learning treats each bundle of waveforms derived from one strain as an aggregated data and utilizes the whole distribution of the strain-based dataset. More sophisticated generalization ability of the AI can be achieved through gathering a greater number of multiple datasets and through improving the AI program.

**Figure 5:**
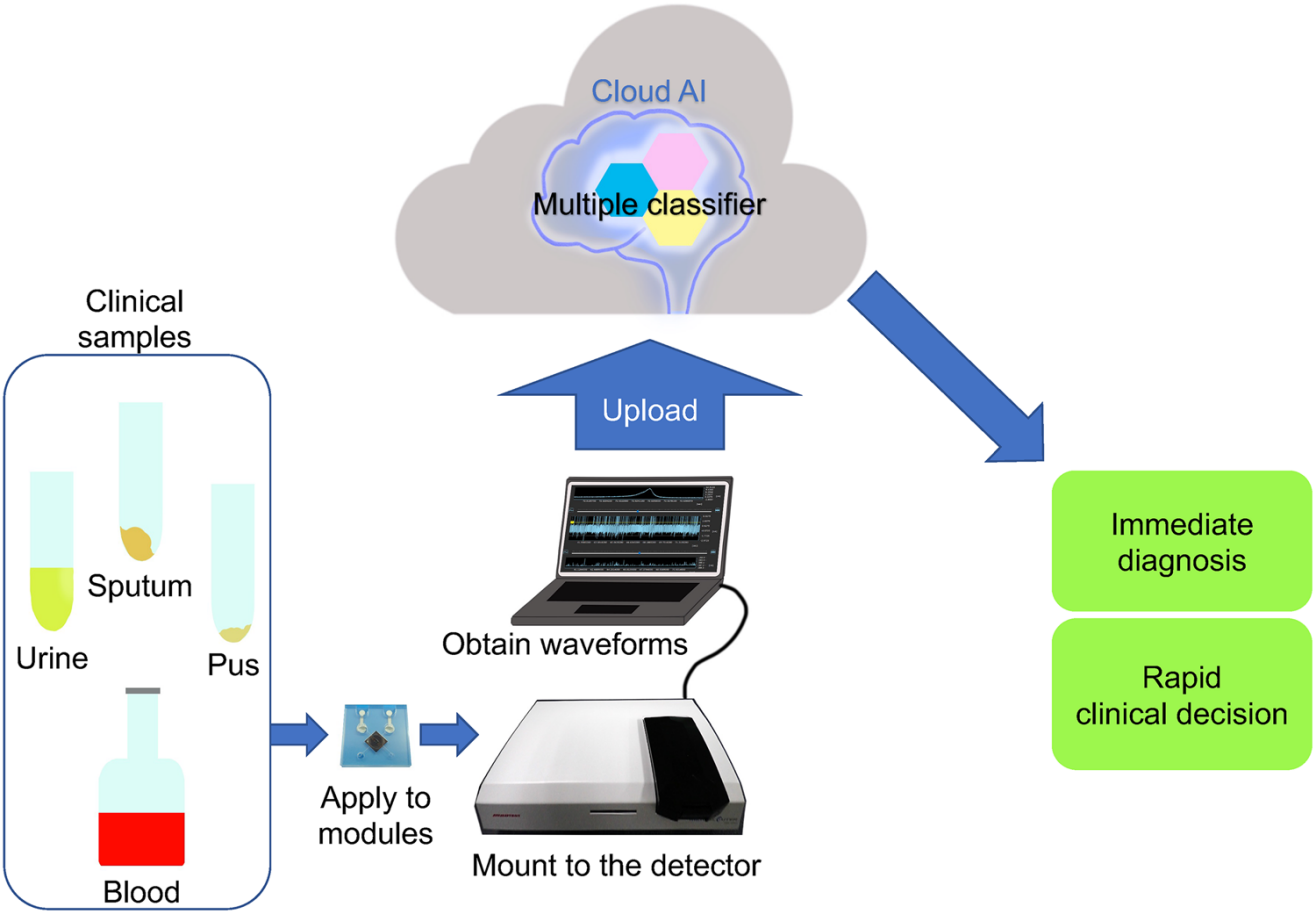
The future perspective of the micropore-artificial intelligence (AI)-based diagnosis. Upon obtaining the clinical samples, they can be analyzed using the modules. The Cloud AI will be able to determine the cause of the infection within a few short minutes.

In conclusion, we developed a novel micropore device combined with machine learning that can distinguish *S. aureus* from *S. epidermidis* with a high sensitivity of 96.4%, independent of existing microbiological identification measures. This technique in which micropore devices and machine learning were combined can significantly contribute to the diagnosis and treatment of bacterial infectious diseases. This development is important, given that it is extremely challenging to create novel classifiers that can widen the range of applications to other types of microorganisms or specimens (e.g., blood, urine, pus, and sputum). Furthermore, ongoing improvement of modules and detectors is likely to help in detecting more subtle differences in the surface structure of bacteria. Our present study is the first step for differentiating clinically isolated bacteria, which leads to the broader approach to other bacterial pathogens and other culture conditions. This micropore-AI combined method can be used as a one-stop bedside platform, to help identify microorganisms from various specimens and their antimicrobial resistance using the internet and the cloud AI system (Fig. 5). Regarding its clinical applications, it is likely that the number of species able to be distinguished and its capability for antibiotic resistance detection will improve.

## Methods

### Preparation of cultured bacteria

We used 50 clinical isolates of *S. epidermidis*, 25 clinical isolates of methicillin-susceptible *S. aureus* (MSSA), and 25 clinical isolates of methicillin-resistant *S. aureus* (MRSA)*,* which were clinically isolated and identified in the laboratory. Methicillin resistance was confirmed by the growth on BD BBL ™ CHROMagar ™ MRSA II Agar Medium (Becton, Dickinson and Company, NJ, USA) and PCR for *mecA* as described by Okuma et al^26^. For differentiating between *S. aureus* and *S. epidermidis*, we used additional ATCC standard strains: ATCC 6538 for MSSA, LAC (USA300, ATCC BAA-1756) and MW2 (ATCC BAA-1707) for MRSA, ATCC 12228 for *S. epidermidis*.

All the isolates and strains were grown in Trypticase Soy broth (Becton, Franklin Lakes, NJ, USA). After overnight incubation at 37 °C, the bacterial cells were washed and diluted in sterile phosphate-buffered saline (Merck KGaA, Darmstadt, Germany), with an optical density between approximately 0.1 and 0.3 at 600 nm (OD600) to avoid clogging of the pores.

### Scanning electron microscopy

We used a JSM-7900F Schottky Field Emission Scanning Electron Microscope (JEOL Ltd., Tokyo, Japan) for the SEM observation of the bacteria.

The bacterial suspensions were prepared for SEM observation according to Rusu et al.’s^27^ previously described protocol, which was modified for our low-vacuum SEM. Briefly, the suspensions were washed three times with PBS. We centrifuged 200 μL of each aliquot at 6000 rpm for 5 min and fixed the pellets with 2.5% glutaraldehyde solution overnight. After fixation, each aliquot was washed three times with distilled water. Subsequently, 10 μL of each aliquot was mounted on a Si wafer and desiccated in a series of ethanol solutions (50%, 70%, 80%, 90%, 95%, and 99.5% for 3 times). After overnight desiccation, we performed SEM analysis. All observations were performed at a pressure of 98 hPa with a 10.0 kV accelerating voltage using a low-vacuum backscatter electron detector.

### Fabrication of micropore modules

A 50-nm- thick SiN film was deposited on both sides of a 0.5-mm- thick silicon wafer by low pressure chemical vapor deposition (LPCVD). SiO_2_ of 300-nm thickness was deposited on top of the SiN film by CVD. Holes with a diameter of 3 mm were drawn by photolithography. SiO_2_ and SiN were etched using reactive ion etching to fabricate micropores. A 1 mm × 1 mm square was drawn on the backside by photolithography. The SiO_2_ and SiN on the backside were then etched using reactive ion etching. Next, Si was etched using KOH. Finally, SiO_2_ was etched with a mixture of HF and NH_4_F. Plastic channels (25 mm × 25 mm × 0.2 mm) were fabricated using polydimethylsiloxane. The Ag/AgCl electrodes were fabricated in the channel.

### Resistive pulse measurements

We obtained micropore-based ionic current–time waveform measurements using a microSCOUTER1200TM (Advantest Corporation, Tokyo, Japan) instrument at a sampling rate of 250 kHz with a 100-kHz low-pass filter. The bacterial solution and 1× PBS buffer solution were placed in the *cis*- and *trans*-side chambers, respectively. The micropores were placed in a special socket, which was then transferred to a measurement device for measurements. A micropore module (base current = 10 nA) was used to measure the bacterial features. To avoid contamination, a micropore module was used for each measurement and disposed of.

### Optical microscopy

An Olympus DSX-1000 optical microscope (Evident Corporation, Tokyo, Japan) was used to capture bacterial movement. A 20× objective lens with 2800× optical zoom was used to observe bacteria in the micropore module. A polarimetric method was used for the observation, and videos were captured at a frame rate of 30 frames per second. A handcrafted fixture was mounted on an optical microscope stage for seating the microSCOUTER 1200™ instrument.

### Machine learning

The ionic current–time waveforms obtained were extracted using commercially available Aipore-PC software (Aipore Inc., Tokyo, Japan) and transferred to the Aipore-OneTM (Aipore Inc.) server. Machine learning was performed using the server. The features were generated using tsfresh. Specifically, the ion current–time waveform was represented by an *N*-divided vector in the vertical direction and an *M*-divided vector in the horizontal direction. As classifiers, we selected the open-source “scikit-learn” classifier and LightGBM. The effects of imbalance in the number of data were prevented using an undersampling method that adjusts the number of data to match the smallest number of data. Classifier training was performed on the combination of generated features and classifiers. The machine learning program outputs the *F*-value, an accuracy measure that can distinguish between two bacteria in a single waveform. The “trained” classifier with the most accurate combination of features and classifiers then identified a particular bacterium belonging to one of two species. The accuracy was assessed through a tenfold cross-validation process. Aipore-ONE™ automatically generates and displays a confusion matrix that assigns one waveform to each bacterial species. The bacteria were then identified on a strain-by-strain basis. Several ion current–time waveforms were obtained by measuring a single strain. Using the classifier and features obtaining the highest accuracy for one ion current–time waveform, the identification results of all ion current–time waveforms obtained during the three-minute measurement were assembled to create a classifier for single strain identification. The single strain classifier was then employed for strain-by-strain testing. Strain-by-strain identification was also performed using Aipore-ONE™.

### Surface charge measurements

The surface charges of the bacteria were measured using a Zetasizer™ (Malvern Instruments, UK). 10 isolates of the *S. aureus* and 10 isolates of the *S. epidermidis* were tested. The bacterial solutions were diluted 100-fold in ultrapure water and placed in standard cells. We performed the measurements for five times per bacterial solution for 120 s at 25 °C. The zeta potentials obtained were statistically assessed using a linear mixed-effects model.

### Statistical analyses

We used the linear mixed-effects model to compare the zeta potentials between *S. aureus* and *S. epidermidis*. All the data measured above were analyzed. We have considered strain level as random effects in the model.

### Supplementary Information

Supplementary Information.

Supplementary Video 1.

## Data Availability

The measurement data of the current–time profile of bacteria obtained in this study are available on Zenodo (10.5281/zenodo.7882378 and 10.5281/zenodo.10547661).
