# Supplementary material for: Using novel micropore technology combined with artificial intelligence to differentiate Staphylococcus aureus and Staphylococcus epidermidis
Source: Sci Rep. 2024 Mar 24;14:6994. doi: 10.1038/s41598-024-55773-4 (PMC10961322; doi:10.1038/s41598-024-55773-4)
Supplement: Supplementary file 1 — Supplementary Information. [file 41598_2024_55773_MOESM1_ESM.pdf]

Using novel micropore technology combined with artificial intelligence to differentiate *Staphylococcus aureus* and *Staphylococcus epidermidis*

Ayumi Morimura[1], Masateru Taniguchi[2], Hiroyasu Takei[3], Osamu Sakamoto[3], Norihiko Naono[3], Yukihiro Akeda[4], Daisuke Onozuka[5], Jumpei Yoshimura[6], Kazunori Tomono[7], Satoshi Kutsuna[1,5,8,9], Shigeto Hamaguchi[8,9,10*]

[1]Department of Infection Control and Prevention, Graduate School of Medicine, Osaka University, 2-2 Yamadaoka, Suita, Osaka, 565-0871, Japan

[2]The Institute of Scientific and Industrial Research, Osaka University, 8-1 Mihogaoka, Ibaraki, 567-0047, Osaka, Japan

[3]Aipore Inc., 26-1 Sakuraoka-cho, Shibuya-ku, Tokyo, 150-8512, Japan

[4]Department of Bacteriology I, National Institute of Infectious Diseases, 1-23-1 Toyama, Shinjuku-ku, Tokyo, 162-8640, Japan

[5]Department of Oral Microbe Control, Graduate School of Medicine, Osaka University, 2-2 Yamadaoka, Suita 565-0871, Osaka, Japan

[6]Department of Traumatology and Acute Critical Medicine, Graduate School of Medicine, Osaka University, 2-15 Yamadaoka, Suita, Osaka, 565-0871, Japan

[7]Osaka Institute of Public Health, 1-3-3 Nakamichi, Higashinari-ku, Osaka, 537-0025, Japan

[8]Division of Infection Control and Prevention, Osaka University Hospital, 2-15 Yamadaoka, Suita, Osaka, 565-0871, Japan

[9]Division of Fostering Required Medical Human Resources, Center for Infectious Disease Education and Research (CiDER), Osaka University, 2-2 Yamadaoka, Suita Osaka, 565-0871, Japan

[10]Department of Transformative Analysis for Human Specimen, Graduate School of Medicine, Osaka University, 2-2 Yamadaoka, Suita, Osaka, 565-0871, Japan

*Corresponding Author: Division of Infection Control and Prevention, Osaka University Hospital, 2-15 Yamadaoka, Suita, Osaka, 565-0871, Japan

Phone: +81-6-6879-5093; Fax: +81-6-6879-5094

Supplementary material

The captured video of the actual screen during the measurement of the ionic currents of the bacteria. The optical microscopic image is shown on the left side, and the waveforms of ionic currents are shown on the right side.
